# Investigating the acceptability and validity of a novel VR paradigm that simulates auditory hallucinations

**DOI:** 10.1016/j.ijchp.2026.100694

**Published:** 2026-05-28

**Authors:** Donagh Seaver O'Leary, Pat Mulvaney, Laura Moore, Lera O’Connor, Brendan Rooney, Keith Gaynor

**Affiliations:** School of Psychology, University College Dublin, Dublin, Ireland

**Keywords:** Virtual reality, Psychosis, Validity, Acceptability, Auditory hallucinations, Psychotic-like experiences, Heart rate variability, Mixed-methods

## Abstract

There is a growing use of Virtual Reality (VR) technologies in psychosis research and intervention. Validated VR simulations of psychotic-like experience have the potential to allow controlled manipulation of stimuli, reproducibility, and to isolate mechanisms of change in an ethical research framework. Although initial developments appear to be safe, qualitative reporting of participants’ experiences of VR simulated psychotic-like experiences is lacking. This paper proposes and demonstrates an ethically grounded framework for evaluating the acceptability and validity of an immersive VR experience that simulates auditory hallucinations (VR-sAH) based upon the prototypical content of auditory hallucinations described by people with lived experience. Sixty-eight non-clinical participants undertook an experimental trial involving VR-sAH. Validity of the VR-sAH was assessed by combined assessment of qualitative reports and psychophysiological responses. Non-acceptability was operationalised as drop-outs, indications of distress during the experiment, and self-reported Subjective Units of Distress post-experiment. Following the experiment, a subset of participants (n = 29) undertook semi-structured interviews guided by the Theoretical Framework of Acceptability. Results indicated that the VR-sAH produced a believable and affectively salient analogue of auditory hallucinatory experiences. Statistically significant reductions in heart rate variability validated participants’ self-reported embodied immersion in the VR-sAH. Semi-structured interviews and quantitative acceptability criteria indicated that the VR-sAH was an acceptable research tool. However, this sentiment was subject to important caveats to consider: including participants with psychosis and other mental health issues, and the importance of integrity and transparency on the part of the researcher team. Although VR-sAHs do not replicate the full phenomenology of clinical auditory hallucinations, they may provide ethically manageable and experimentally controllable analogues of perceptual and cognitive processes relevant to psychosis research, particularly during early-stage development in non-clinical populations.

## Introduction

Virtual Reality (VR) technologies are increasingly being used within psychosis research to simulate psychotic-like experiences in controlled experimental settings. Although immersive VR paradigms offer substantial promise for investigating the mechanisms underlying hallucinations and related symptoms, comparatively little attention has been given to the ethical evaluation and acceptability of these tools, particularly prior to their use with clinical populations. The present study investigates the acceptability and validity of a novel VR simulation of auditory hallucinations (VR-sAH) previously developed and utilised by our research group (Seaver O’Leary et al., 2026). This study aims to contribute towards ethically grounded standards for reporting the acceptability of immersive VR psychosis paradigms through the integration of psychophysiological, qualitative, and distress-based measures. Establishing the acceptability of such paradigms in non-clinical populations may provide an ethically necessary intermediate stage prior to implementation in clinically vulnerable groups.

### Virtual reality

There is a burgeoning use of Virtual Reality (VR) technology in psychology (Bell et al., 2024; Kim & Kim, 2020; Riva, 2022; Zeka et al., 2025), considered by some optimistic commentators as “the beginning of a long history” in its application in treatment interventions (Cerasa et al., 2024). Increasingly, VR is being utilised in psychosis research in various ways including the development of VR-simulated analogues of symptoms of psychosis (e.g., Pina-Thomas et al., 2025; Roberts et al., 2024). Psychotic disorders are a heterogeneous group of mental disorders that are characterised by positive symptoms (i.e., delusions and/or hallucinations), disorganized thinking and/or motor behaviour, and negative symptoms (i.e., affective blunting, alogia, asociality, anhedonia, and avolition)(Arciniegas, 2015; Maj et al., 2021). Auditory hallucinations are a common symptom, occurring in approximately 64–80 % of those with primary psychotic disorders (Toh et al., 2025). At sub-clinical thresholds, psychotic-like experiences (PLEs) occur across the general population (Seiler et al., 2020), with estimates indicating that mean lifetime prevalence of psychotic-like experiences range between 5–7 % (McGrath et al., 2015; Staines et al., 2022).

Because alterations in the experience of reality are a defining feature of psychosis, it is a condition that is particularly well suited to VR. In relation to the next generation of psychological interventions for psychosis, leading researchers Nelson et al. (2021) make a case for a more robust sense of “self-presence” in interventions which in turn encourages a greater sense of embodiment - of “being-there” – by incorporating immersive, body-oriented tasks such as physical activity and mindfulness practice into treatment. They suggest that VR can be useful to construct simulated scenarios equivalent to the lived experience of psychosis. Indeed, VR allows for the safe inclusion of analogues of psychotic symptoms (e.g., auditory hallucinations) to be delivered in experimental settings (Ward et al., 2020). Importantly, VR facilitates the controlled manipulation of stimuli, reproducibility of experiments, and the potential to discreetly investigate models of psychosis of in experimental settings. Previous findings suggest that VR-based interventions for psychosis can be effective, safe, and cost-effective (Bell et al., 2024; Kim & Kim, 2020; Pot-Kolder et al., 2020) and that client attitudes towards VR are positive (Bell et al., 2024; Chan et al., 2023). However, there remain few studies involving VR simulated psychotic-like experiences. Until now, such paradigms have been used primarily for pedagogical rather than experimental reasons (e.g., Pina-Thomas et al., 2025; Roberts, Hulme & McCann, 2024; Roberts et al., 2024).

### Ethical considerations

Given that many people with a psychotic disorder experience a disconnection from reality, unique ethical considerations are needed for those with psychosis in order to safeguard any potential adverse of VR technologies (Madary & Metzinger, 2016; Marloth et al., 2020). In their landmark paper proposing best practise guidelines and considerations surrounding the use of VR in psychology, Madary & Metzinger (2016) have argued that the use of immersive VR in research must meet two non-negotiable criteria: (1) interventions must not increase the risk of harm, and (2) their design must anticipate the vulnerabilities of the population they aim to benefit. The seriousness with which psychosis researchers must take these precautions is non-trivial when considering the potential negative impact associated with VR-based interventions for a small number of participants in psychosis intervention (e.g., Garety et al., 2024; Smith et al., 2025). Currently, adverse effects are under-reported, with less than half of studies making any reference to them (Lundin et al., 2023). VR as a tool in psychology is in its infancy and developing so rapidly that it has few precedent cases from which to ground development strategies. This poses a dilemma: in order to set precedents and drive forward ethical research, novel research must be conducted. In order to facilitate this, the establishment of a standardised, ethically sound reporting process for VR tool development is needed. Moreover, until more is known about potential adverse effects of these technologies, clinical populations should be included incrementally.

### Acceptability of the VR-sAH

A well-established framework in which to assess such risks early in intervention development is by investigating its acceptability. Acceptability is a multi-dimensional construct that evaluates the extent to which people providing interventions or receiving them consider them to be appropriate, based upon individuals’ anticipated and experienced attitudes and feelings towards the intervention (Sekhon et al., 2017). Despite many studies highlighting the need to establish acceptability of VR for psychosis, few specifically report such findings (Rus-Calafell et al., 2018). The limited number of published qualitative assessments reveal that patients’ attitude towards using virtual environments are generally positive (Bell et al., 2024). The Theoretical Framework of Acceptability (Sekhon et al., 2017, 2018) has been successfully adapted for VR-based intervention studies in healthcare settings (Storey et al., 2024), as well as recently in the development of a VR-based psychosis intervention by Meins et al. (2025).

### Validity of VR-sAH

Little is known about the realism, immersiveness or believability of a VR simulation of selective features of psychosis symptoms. Psychophysiology allows researchers to study the relationship between mental processes and bodily responses to stimuli, providing insights into how psychological states impact physiological functions and vice versa (Potter & Bolls, 2012). The gathering of psychophysiological data during a VR experience can thus add crucial insights to participants’ experience beyond subjective self-report. Psychophysiological measures are frequently used in studies investigating individual differences in reactions to VR (e.g., Magalhães et al., 2024; Marín-Morales et al., 2021), and cardiac parameters have successfully been correlated with psychological measures in VR studies (Ronca et al., 2024). Cardiac parameters recorded using electrocardiogram (ECG) are a reliable and common way to approximate autonomic function (Pham et al., 2021; Potter & Bolls, 2012). Heart Rate (HR) is the number of heart beats per minute; it rises due to the influence of the sympathetic nervous system (SNS) and decreases with the control of the parasympathetic nervous system (PSNS). Heart Rate Variability (HRV) is the variation in time between successive heartbeats and can detect more subtle autonomic nervous system (ANS) changes compared to HR (Shaffer & Ginsberg, 2017). A decrease in HRV is connected to increased Sympathetic Nervous System (SNS) activation (and/or reduced PSNS) and an increase in HRV to PSNS control (and/or decreased SNS) activity (Potter & Bolls, 2012; Shaffer & Ginsberg, 2017). HRV time-domain measures are derived from calculating statistical values directly from the sequence of interbeat (R-R) intervals.

### Research aims

The aim of this research is to investigate the acceptability and validity of a novel VR paradigm that includes a simulation of auditory hallucinations (VR-sAH) using quantitative, qualitative and psychophysiological data. Specifically, the study aims to evaluate whether a VR-sAH paradigm can be implemented in a manner that is both experientially valid and ethically acceptable within a non-clinical population, while contributing towards more standardised reporting practices for immersive VR psychosis research.

## Method

### Overview of study design

Sixty-eight non-clinical participants undertook an immersive virtual reality experience that included a simulated analogue of auditory hallucinations (VR-sAH). The study utilised cardiac parameters (measured using ECG) as a validity measure to assess autonomic arousal and to indicate that the participants found the task emotionally salient and cognitively demanding. After completion of the experimental task and the post-experiment questionnaires, a semi-structured interview took place with a random subset of participants (*n* = 29, 43 % of total sample) to explore their experience of the VR-sAH for validity and acceptability. Qualitative analysis of interview data was conducted using Thematic Analysis (Braun & Clarke, 2006). Acceptability was also assessed by examining drop-out, distress during the task and Subjective Units of Distress (SUD).

#### Power analysis

An a priori power analysis was carried out using G*Power (Faul et al., 2007), paired samples t-test, showing that a sample size of 71 could detect a small-medium effect size (*d* = . 3) in outcome measures with a significance level of *p* = .05.

### Participants

#### Inclusion/Exclusion criteria

Due to the novelty of the experimental design, stringent constraints regarding recruitment were used. Drawing on these ethical considerations, participants were required to meet the following criteria in order to participate: (a) between 18–70 years of age, (b) English speaking, (c) no other language difficulty (i.e., difficulty speaking, listening or reading language), (d) no history of psychiatric disorder; (e) no history of any neurological disorder (including photosensitive epilepsy); (f) no history of, or current, substance abuse; (g) no intellectual impairment (e.g., intellectual disability or neurodegenerative disease); (h) no hearing or vision deficiencies; (i) currently taking no psychiatric medication (e.g., antipsychotics, antidepressants, benzodiazepine). These requirements were self-declared by participants.

The use of non-clinical participants in studies investigating psychosis-related phenomena is well-supported by evidence suggesting that psychotic-like experiences, such as unusual perceptual experiences, magical thinking, and paranoid ideation, exist on a continuum in the general population (Linscott & van Os, 2013; Peters et al., 2016; Ward et al., 2020). This dimensional perspective allows researchers to examine subclinical expressions of psychosis without the confounding influences of medication, hospitalisation, chronic illness, or diagnostic uncertainty (Mason, 2015).

#### Recruitment

A convenience sampling methodology was used. Sixty-eight non-clinical participants were recruited via posters in Dublin, Ireland and digital recruitment posters. Some participants were recruited via University College Dublin’s (UCD) SONA system; an online participant recruitment platform that allows psychology students to partake in research experiments within their school to earn research credits that can be used towards recruiting participants for their own future research. No participants were remunerated for their time.

#### Ethical considerations

This research was conducted in accordance ethical standards laid out in the Helsinki Declaration (World Medical Association, 2025). It received full ethical approval from the host institution’s Human Subjects Research Ethics panel (HS-24–90-OLeary-Gaynor).

#### Demographics and experimental preparation

Socio-demographic variables, self-reported cardiovascular exercise frequency and CAPE-42 (Stefanis et al., 2002) scores were collected pre-experiment using the online software Survey Monkey. The CAPE-42 is a widely administered self-report scale consisting of 42 items that assesses positive, negative, and depressive symptoms in relation to psychotic experiences in the general population (Supplementary Material B).

In order to obtain optimal HRV data, all participants were issued with advance instructions prior to the experiment in line with best practise guidelines set out by Quintana and colleagues (Quintana, 2017; Quintana et al., 2016; Quintana & Heathers, 2014). Participants were instructed to adhere to the following before their arrival: (a) refrain from ingesting alcohol or drugs for 24 h prior; (b) refrain from ingesting caffeine products for 2 h prior; (c) refrain from drinking any liquids in excess of 500 ml for 1 h prior; (d) refrain from eating a large meal 1 h prior and, if possible, to have a light meal about 2 h prior; (e) refrain from using nicotine products for 30 min prior. Upon arrival to the laboratory participants were encouraged to use the toilet facilities if needed.

### Materials

#### ECG

ECG recording was undertaken with a Powerlab 26T (ADInstruments, Dunedin, New Zealand) and using LabChart software (v.8.1.29 for Mac, AD Instruments, Dunedin, New Zealand). A sampling rate of 4k/s was set, and incoming signals were underwent filtering using a 20 Hz Low-pass filter, 0.05 Hz High-pass filter, mains filter, and anti-alias filter. The disposable electrodes used were standard Ag/AgCl sensors and connected via snap-connect lead wires to a Bioamp cable (ADInstruments, Dunedin, New Zealand).

#### Virtual reality

**Fig. 1:**
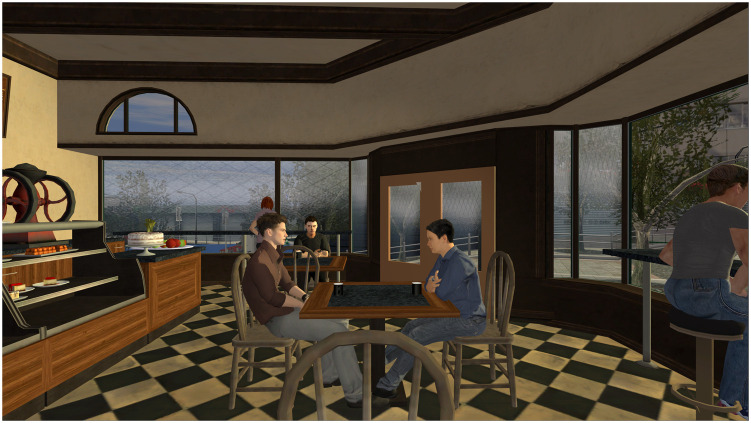
VR experience from the perspective of the participant.

The immersive VR scenario was run on a Lenovo Desktop with an Nvidia GTX1070 graphics card. Participants wore a head-mounted Meta Quest 3 (Meta, USA) with a display resolution of 2064 × 2208 pixels per eye and a max Frame Rate of 72fps. This research built upon a previously developed tool developed in Unity called *Coffee Without Words* that generates a scenario whereby the participant is sitting stationary in a busy café (Fig. 1). Participants were free to visually search the simulation by moving their heads around as much or as little as they wanted. The café included various virtual avatar bystanders and ambient sounds of chatter that would be typical of this setting in the real world. One of the male avatars, seated a few tables away, intensely stares at the user intermittently. This tool was specifically designed based on empirical research to probe social interactions. It has previously been used to investigate social cognition in a naturalistic setting (e.g., Rooney et al., 2017). During the immersive VR experience, participants heard the Hearing Voices Simulation (Supplementary Material A). This audio track - designed by individuals with schizophrenia - replicates the stereotypical content of an auditory hallucination that would be experienced during psychosis. The audio of the Hearing Voices Simulation was projected by the headset’s speakers so as to be perceived as having an external source for the participant.

### Measures

#### Quantitative measures

2 min of ECG data were produced for each of Baseline and the VR-sAH condition. Heart Rate (HR) was operationalised as beats per minute (BPM) and Heart Rate Variability (HRV) was operationalised as the root mean square of successive differences between heartbeats(RMSSD). Distress for all participants was quantitatively recorded and logged in the in the following ways: dropouts, participants indicating distress in-session, Subjective Units of Distress >80/100 measured by self-report post-experiment.

#### Qualitative measures

A sub-set of participants were invited to complete an exit interview after the experiment was concluded. Simple randomisation using a computer-generated random number sequence was used for this process. Following the principles of Thematic Analysis (Braun & Clarke, 2006), interviews were ceased after 23 participants (43 % of total sample) as saturation (the point at which no new insights or information was brought up in interviews) was evident.

The exit interview exploring the perceived acceptability of the VR-sAH was developed based upon Sekhon, Cartwright and Francis’s (2017) Theoretical Framework of Acceptability and subsequent questionnaire recommendations (Sekhon, Cartwright & Francis, 2022).These 4 open-ended questions were followed up with further inquiry to explore participants’ reflections on the VR-sAH (e.g., “can you tell me more about that?”). All interviews lasted between 5–12 min. Interview schedule: (i) **General Acceptability.** How acceptable was the VR experience for you, overall? (ii) **Perceived Effectiveness.** Do you think the VR experience achieved simulating what it is like to experience hallucinations? (iii) **Affective Attitude**. How comfortable did you feel *during* the VR experience? (iv) **Ethicality.** How fair do you think the VR experience is for people? Do you think that there are moral or ethical considerations that need to be addressed?

### Procedure

Prior to the immersive VR experience, baseline ECG measures were taken for 2 min. Then, wearing a Meta Quest 3, participants experienced the VR-sAH for 2 min, during which time ECG measures were recorded. The full description of the experimental procedure is provided in Supplementary Material C.

### Data reduction and analysis

#### Quantitative data analysis

The full description of data cleaning, preparation and statistical analysis is provided in Supplementary Procedure D.

#### Qualitative data analysis

Audio of the interview was recorded using the Voice Memos app on a 2020 MacBook Pro (Apple, USA). The audio was then transcribed using Descript software. The resulting transcript files were then imported into NVivo where Thematic Analysis (Braun & Clarke, 2006) was employed to analyse the data. In order to assure inter-coder reliability, data were first coded separately by the three researchers (DSOL, LM and LOC) and formed into themes. Then, the three qualitative reports were consolidated into a single thematic structure that best represented the interview data. Final decisions on what was included were taken by the lead researcher (DSOL).

#### Reflexivity statement

Analysis was shaped by active reflexivity throughout the research process; in data collection, analysis, and interpretation. The lead author is a postgraduate researcher investigating psychosis and was aware of his position within this realm of knowledge production. The two additional authors were lab interns with psychology backgrounds, who had received training in Thematic Analysis. The researchers’ own biases were acknowledged and held in constructing the interviews, in dialogue with participants, and throughout the analytic and writing stages. The researchers attempted to remain impartial, non-judgmental, grounded in an ontological perspective of critical realism.

## Results

### Missing data

There were no missing data for demographic variables. Due to a technical error, one participant displayed missing data for all cardiac parameters and was excluded from analysis. Of the sub-sample that completed exit interviews (*n* = 29), all participants answered each interview question.

### Sample characteristics

Sixty-seven participants were included in the final quantitative analysis. Demographic characteristics and CAPE-42 scores are presented in Table 1. Both CAPE-42 total and CAPE-42 positive symptom subscale scores indicated that overall the participant sample showed low psychosis proneness. The mean weighted positive symptom subscale score (the sum score of 20 subscale items divided by number of items) was 1.33, indicating that the study sample reflects other non-clinical European student populations CAPE-42 scores (e.g., Netherlands = 1.37, Norway = 1.31) (Vermeiden et al., 2019).

**Table 1:** Characteristics of study sample.

Variable		
Age, *M* (*SD*)		29.18 (12.55)
Sex, *N* (Male/Female)		27 M, 40F
CAPE-42, *M* (*SD*)		64.26 (10.91)
Cardiovascular activity, *N* (%)		
	Every day	7 (10.3)
	A few times per week	36 (54.4)
	A few times per month	19 (27.9)
	< Once per month	2 (2.9)
	Never	3 (4.4)
Nationality, *N* (%)	Irish	47 (70.6)
	Other European	10 (14.7)
	North American	4 (5.9)
	South American	1 (1.5)
	African	1 (1.5)
	Asian	4 (6)
Education, *N* (%)		
	Leaving Cert	4 (5.9)
	Some College, No Degree	20 (29.4)
	Higher Diploma	2 (2.9)
	Bachelor’s Degree	17 (25)
	Postgraduate Degree	24 (36.8)

Note. N = 67.

### Quantitative results

#### Distress

There were no participant dropouts during the experiment, and no participants verbally indicated distress at any stage of the procedure. Post-experiment Subjective Units of Distress (SUD) scores following the VR-sAH were low overall (M = 19.87, SD = 16.35, Md = 15, range = 0–70). No participant reported a SUD score exceeding the predetermined threshold of 80 for significant distress.

#### Cardiac parameters

Paired samples t-test were carried out to examine the effect of the VR-sAH on cardiac parameters (HR, RMSSD)(Table 2). Due to violations of assumptions of normality, RMSSD scores were log (ln) transformed for analysis. The log (ln) transformed RMSSD scores met assumptions of normality. There was no statistically significant difference in participants’ HR scores between Baseline and the VR-sAH condition. There was a statistically significant difference in participants’ ln(RMSSD) scores between Baseline and the VR-sAH condition. The mean increase in ln(RMSSD) scores was 0.12 (95 % CI: 0.02 to 0.22 ); this indicated a small-medium effect, Cohen’s *d* = 0.3.

**Table 2:** Paired sample t-test results for cardiac parameters.

Variable	Baseline *M* (*SD*)	VR-sAH *M*(*SD*)	t (df)	*p*	*d*
HR	68.09 (16.66)	68.37 (14.2)	−0.41 (1, 66)	.68	-
ln (RMSSD)	3.82 (0.66)	3.69 (0.6)	2.47 (1, 66)	**.01**	.3

*Note. N* = 67. HR = Heart Rate, RMSSD = Root Mean Square of Successive Differences. *d* = Cohen’s *d*. Text in bold font indicates statistically significant findings.

### Qualitative results

Using Thematic Analysis, themes for each Research Question were constructed that best represented participants’ reflections on the VR-sAH paradigm, based upon four pre-determined Acceptability constructs. These themes are displayed in Table 3. Participant quotes are all pseudonymised by their unique study ID (e.g., P23, P45, etc.).

**Table 3:** Description of Themes.

Category	Theme	Quote
1. General Acceptability	1a. Fostering Curiosity and Empathy	"I think it's cool to put yourself in that situation and like to actually maybe experience a tiny bit of what people with psychosis actually experience themselves”
	1b Striking the Right Balance	“it wasn't pleasant, but it wasn't going anywhere further than that, you know?”
	1c. Milder Than Expected	“I felt very comfortable. Probably a little too comfortable”
2.Perceived Effectiveness	2a. Lack of Lived Experience	“I always kind of wondered like, do you, when you're hearing voices, do you think it's like, it, does it feel more like auditory stimulus or does it feel like it is like inside thoughts?”
	2bDesign Considerations	“when I first heard it, I was like, I was like, kind of like, I turned my head a bit because I was like, oh, it's not like actually you know, happening in this, like, is there a woman there?”
3. Affective Attitude	3a. A Range of Experiences	“I was expecting more intense audios […] that was quite, quite comfortable and yeah, reasonably relaxing to be honest!”
	3b The Influence of Expectations	“I thought that they would maybe be a little bit more scary or worse”
4. Ethical Concerns	4a. Something of Potential Value	“the research on this is going to going to help people who, for whom this is a reality, not just a, a, a sort of VR experience”
	4b Considering those with Mental Illness	“I can only see concerns for people who might actually be hearing voices”
	4c. The Responsibility of the Research Team	“what made it acceptable was the fact that you had alerted me to the fact that there was nothing scary going to happen”

#### General acceptability

Encouragingly, all participants contended that this immersive VR experience was overall an acceptable experimental paradigm.

##### Fostering curiosity and empathy

For some, the experience fostered a sense of empathy and curiosity towards those living with psychotic symptoms:

I think it's cool to put yourself in that situation and like to actually maybe experience a tiny bit of what people with psychosis actually experience themselves. Um. So I think it's, yeah, I think it's a fairly interesting educational experience for people rather than anything that would be distressing … I find it very interesting (P41)

Reflecting on her experience, P36 was compelled to think that living with voice hearing "would definitely be disconcerting if this happened all the time” and it "would be terrifying to have to hear all the time and like really would not make me feel good”.

##### Striking the right balance

It was considered by many that a suitable balance had been struck between the VR experience being stressful, yet tolerably so, with “nothing too graphic to be seen or heard” (P11):

it wasn“'t pleasant, you know, it wasn'”t pleasant, but it wasn't going anywhere further than that, you know? It wasn't like, 'oh God, I don't wanna be in this anymore.' It's like, I'm totally calm and collected, but that's a bit unpleasant (P25)
I had a feeling the voices would be like scarier or something, whereas they weren't too bad (P28)

Striking such balance was considered significant for the overall acceptability. An interesting thread emerged whereby some participants considered the non-specificity of the content of the Hearing Voices Simulation (HVS) (i.e., that the audio recording is a generic track that each participant heard) to be a good thing because it limited the potential for negative reactions to targeted negative or disparaging voices. For some, it was specifically a concern that any simulation of hearing voices that addresses an individual’s insecurities may be problematic:

I don't think that the content of what's being said is too specific or unique, to me at least, to cause me like, overt insecurity or like, worry. Like, that sort of hearing the voices in your head and spiralling into sort of negative thoughts would be more intense or upsetting if it was, like, tailored to me. Obviously it can't be tailored to you, but if it was, like, it could be more, it could resonate more with specific people depending on, like, the content (P18)

Tellingly, this reflected P55’s own experience. She at one stage found the HVS content to be unsettlingly referential:

Um, but that was like one point I can't quite remember what they were saying. Where it was like quite jarring 'cause it felt like they were talking to me. About me. And I was like, 'oh, relax’ […] I can’t remember what they said, but it sounded like, it was like, 'how did you know that about me?', type of thing. It's like, ‘why do you know that?’. Because there is something about like self and, something I can't remember. Um, and I was like, 'don't say that about me!’ (P55)

##### Milder than expected

The interviews revealed a wide range of individual appraisals of the degree of perceived distress. P70 and P73 both reported feeling “pretty comfortable” throughout the experiment. P74 described the process as “really relaxing”. Indeed, many found the entire experience underwhelming compared to expectations:

No, yeah, I found it completely acceptable, quite um, yeah, a lot milder than I was expecting to be honest. (P13).
I felt very comfortable. Probably a little too comfortable. I probably could have, I felt I could have done with being a little bit more in the, in the experience. (P1)

#### Perceived effectiveness

##### Lack of lived experience

The most common caveat to participants’ considerations about the perceived effectiveness of the VR tool was that none of them had lived experience of hearing voices. An equivocal ‘yes’ was the universal answer (“it *seemed* quite realistic”, P13). Although participants were all familiar with the concept of hearing voices, the exact qualities of such an experience were mostly unknown to them:

I always kind of wondered like, do you, when you're hearing voices, do you think it's like, it, does it feel more like auditory stimulus or does it feel like it is like inside thoughts? (P36)

This lack of lived experience had a greater impact than merely not facilitating definitive opinion to the interview question. P1 reported feeling reduced sense of immersion to the VR because he didn't feel that it was what it would be like to hear voices:

Yeah, I have no idea what it's like to hear voices, so. But in my head, I hear splintered rather than sentences. I hear, I hear, I definitely hear Irish voices rather than an American voice. I'm not sure I hear a female voice if I'm hearing voices. I don't know, is the answer, but for me, this is my prejudice (P1)

Nonetheless, many were able to extrapolate their own lived experience onto the situation. In general the content was considered to resemble a plausible accuracy of auditory hallucinations:

But, uh, yeah, it seemed, seemed fairly accurate, you know, like it was reasonably well timed with the person like two tables over kind of looking up and yeah, it seemed quite realistic as to how, you know, uh, someone who's neurotic would kind of, their thoughts would play it, and it seems quite, quite realistic. (P13)

##### Design considerations

While participants admittedly lacked a frame of reference, positive attitudes towards both the VR hardware and software increased the perception of it achieving an accurate simulation voice hearing. The panned projection of the voices within the simulation (i.e., that they sounded as if their source was shifting from left ear to right ear, at varying distances from the participant) was considered to have created a greater sense of realism. P37 describes how this prompted continuous appraisal of her situation:

So it's kind of like sometimes it would, I'd be like, is that like that person over there saying it or is this in my head? And sometimes. It was kind of just like background chatter. Like you were at a busy train or something like that (P37)

Perhaps the most informative clue as to the perceived effectiveness of the VR simulation was the embodied engagement that many reported. In response to the perceived external voices, participants found themselves physically orienting their heads towards the anticipated source: "your instinct to kind of check if the people in the room were speaking” (P37), “I nearly thought [the voice] was behind me, so I started looking behind me at some point” (P76) . When neither source nor recipient could be attributed to the voices, the realisation emerged that this was analogous to the experience of hearing voices. P2 noticed themselves looking around in trying to understand the target of the voices that she heard: "And then I realised it was *me*” (P2).

when I first heard it, I was like, I was like, kind of like, I turned my head a bit because I was like, oh, it's not like actually you know, happening in this, like, is there a woman there? Um, so in that sense it's like, um, it didn't like, I guess like, I dunno, you could argue like, I, I, what I thought I heard was just a different person. So it wasn't that I was hearing in my, in my head, not necessarily. 'cause I just felt like it was, I was hearing it through my ear. But then you then, like, as you're going on and the, the, the content that's being said, you go, oh yes, some of this is hap- this is happening inside of me. And it's about me. And then you're like, yeah, okay. This is, I understand now that this is like thoughts in my head (P25)

Additionally, the immersive nature of the café scenario appeared to add to the realism of hearing voices for P41:

I found like you were kind of caught up in, in the context of the cafe and then, you know, these voices would kick in[…..]Um, so yeah, I thought, I thought it did it well (P41)

For P2, her immersion was hampered by the low fidelity of the visual signal:

I found the, um, the blurriness, um, I, I would have found it more convincing had there been sharper images. Um, because at one stage I was struggling to see, um, eye movement. . Um, and if there had been sharper features […] it would have made it more real (P2)

For others, the low fidelity of the VR café hindered their ability to connect with the experience, at times feeling as if they were “watching something on telly although I was supposed to be in it” (P82):

This was, for some, offset by the realistic nature of the Hearing Voices Simulation (HVS): “Visually, it doesn’t look particularly realistic […...] but when the audio does start you’re kind of caught off guard” (P66). For others, “the voices in my head didn't seem like real voices. I kind of felt like I was watching a movie ”(P72). Tellingly, there was a wide variety in how immersive participants’ found the experience. For example, P68 stated that using VR to convey a certain environment was “better than just staring at a flat screen”, and P72 said that “it *seemed* like it was all around me.”

#### Affective attitude

##### A range of experiences

A spectrum of experiences were reported, from moderately uncomfortable to perhaps not uncomfortable enough. The majority reported that the experience was neither too negatively nor positively valenced. Broadly, the experience was appraised as "relatively comfortable” (P18), "a bit weird at first” (P28), if not a "bit tense” (P55):

I mean, it, it was, it was like, there was, there was no stage in it where I was like 'get me outta this'. Like I was totally fine to sit there and like, [experience it for] way longer, like it was, it wasn't causing anything like actual like fight or fight response or anything like that. But yeah, it was just mildly unpleasant at some points (P25)

Towards the more distressing pole, the entire uncanny experience (including the HVS, the VR world itself and the strange sensation of being disembodied) was for P37 “unsettling. […] Yeah, that's, that kind of sum- sums it."

However, several participants reported the experience as underwhelming:

I was expecting more intense audios and kind of more intense visuals, so I think my expectations maybe tempered the reality. But yeah, that was quite, quite comfortable and yeah, reasonably relaxing to be honest! (P13)

##### The influence of expectations

What emerged within the conversations was that the expectations of participants prior to the experiment appeared to be a significant factor framing their affective attitude during the VR experience. These expectations need not have been as grave as dread or trepidation, but as P28 phrased it: "maybe like anticipation. I'm not sure exactly what it was gonna be like”. Many reported that their pre-appraisal of what they were about to experience greatly coloured the experience itself:

Um, well, no, it's just that, um, I don't, I don't suffer, I've never had any hallucinations, so I don't know what those sound or look like, so, um, um. So I thought that they would maybe be a little bit more scary or worse, but I think that was fine. (P11)

For P37, what he brought to the experience meshed with the inherent discomfort of the VR experience. Immediately he began to have doubts as to whether he was mentally prepared:

am I going to be able to handle this? […] Uh, and that kind of, you know, that it wasn't going to unlock something in me. Do you know what I mean? […] Now neither of them were like an unbearable, uncomfortable uncomfortableness. But it was like kind of low lying, kind of two-pronged uncomfortableness (P37)

#### Ethical concerns

##### Something of potential value

Promisingly, no participant conveyed that this experimental design was ethically problematic. Indeed, some explicitly expressed that the development of such a tool in aid of research is a valuable goal, even if the cost/gain balance must be weighted carefully:

I think it's very necessary because moving forward, if, if, you know, the research on this is going to going to help people who, for whom this is a reality, not just a, a, a sort of VR experience. I think it's a great idea to have, you know, to expand that, um, in order, you know, because the only thing that can come out of this is good. So I think it's a very, very worthwhile for the researcher to do. (P2)

##### Considering those with mental health conditions

However, this stance came with admonitions regarding who participates in future experimental trials. Strong opinions as to the need to exercise prudence in developing this research tool were expressed by many. To quote P8, the VR tool "is something I think that needs to be used with caution.” Of particular concern was the morality of exposing those with mental health difficulties to the VR experience, "to take into consideration a person's background. If they are more prone to experiencing this in real life” (P11): “for serious psychiatric conditions, I would be hesitant” (P28). P25’s own lived experience with social anxiety made him sensitive to the negative potential of the VR tool:

Just like, yeah, because it is like I, I've, in my past I've dealt with social anxiety. and like the thoughts, the things that were said were like similar to certain things that I would be saying to myself. So just for that reason, I can see that potentially being something that could heighten in someone who is like currently dealing with that kind of stuff

Given the ad hominin, disparaging remarks of the voices that they heard, some participants raised potential ethical concerns for those who have experienced psychological or emotional abuse: “That it didn’t trigger something like that for someone, that what is being said isn’t something that was said to a person as a child. That could be quite triggering” (P82).

##### The responsibility of the research team

These concerns notwithstanding, there was general agreement amongst participants that once stringent best practise guidelines are adhered to, there should be little need for concern. This inherent trust in the responsibility of the research team was evident as many referenced the prior information informing their experience. Trust that there would be nothing untoward or unexpected included in the VR paradigm was crucial for participants’ attitude of acceptability:

what made it acceptable was the fact that you had alerted me to the fact that there was nothing scary going to happen and that I was going to hear voices … For me, you prepared me well for what I saw, so it was very acceptable. (P1)

## Discussion

The aim of this research was to investigate the acceptability and validity of a novel VR paradigm simulating auditory hallucinations (VR-sAH), while also contributing to the development of ethically grounded reporting standards for immersive psychosis research. The present findings support the utility of VR-sAH paradigms within non-clinical populations as an intermediate and ethically cautious stage in the development of immersive psychosis research tools.

### Validity

The validity of the VR-sAH was assessed in two ways: psychophysiological responses and qualitative data from the exit interviews. Results from the analysis of cardiac parameters revealed that VR-sAH produced a change in heart rate variability (HRV)(interpreted from log transformed RMSSD scores) but not heart rate (HR). The decrease in RMSSD scores indicates a predominance of sympathetic tone, given HRV’s correlation with autonomic arousal (Kim et al., 2018; Potter & Bolls, 2012). This indicates that participants experienced the VR-sAH as emotionally salient and cognitively demanding. Although the effect size was small-medium, this is comparable to other psychophysiological studies where small effect sizes are generally reported (Quintana, 2017). HRV changes in relation to stress, corresponding to the magnitude of the stressor (e.g., anaerobic exercise produces a much larger increase than listening to music). Media exposure, by design, will generally not increase HR or HRV to a large degree; small effect sizes are normal (Quintana, 2017).

In addition to the psychophysiological evidence, interview data revealed that participants engaged with the VR-sAH in a manner encouraging for research validity. A tell-tale sign of this was the reported embodied, orienting movements as evidenced by the head turning in response to the voices (2b Design Considerations). This speaks to the value using such VR tools as a way of delivering experimental stimuli with high immersion and the realistic experience of embodiment. This is important because what distinguishes VR from other media is ‘presence’: the psychological feeling immersed in the virtual world instead of observing it. In general, a VR technology is immersive when it is able to sensorially separate the user from the physical world and to replace his/her sensory stream with the simulated scenario generated by the computer; the disappearance of the medium from conscious attention (Riva, 2022).

### Acceptability

Acceptability of the VR-sAH was assessed in two ways: logging distress and qualitative data from the exit interviews developed using the Theoretical Framework of Acceptability (Sekhon, Cartwright & Francis, 2017). Quantitative measurements of distress indicated that this was an acceptable research paradigm. No participants withdrew from the study or verbally indicated distress during the experiment. Post-experiment SUD scores remained low overall, and no participant reported a score exceeding the predefined distress threshold of 80, supporting the acceptability of the VR-sAH paradigm.

Interviews indicated that the VR-sAH was considered universally acceptable by participants (1. General Acceptability). However, many contended that the acceptability hinged on the basis that they experienced a reasonable level of distress, which in this case was considered to be within tolerable bounds (1b Striking the Right Balance). However, it was notable that across participants, there was a wide range of experiences reported (3a. A Range of Experiences). Some found it tame (“relaxing”) while others experienced mild to moderate discomfort (1c. Milder Than Expected; 3a. A Range of Experiences). Individual differences regarding past mental health history appeared to have an effect on responses (4b Considering those with Mental Illness).

The most strongly held belief articulated in interviews was that a possible ethical issue would arise by heedlessly including clinical participants in future VR-sAH studies, particularly those with psychosis (4b Considering those with Mental Illness). An important consideration is that most participants had a limited knowledge of the nature of psychosis and its lived experience. Many specifically acknowledged this in interviews, expressing uncertainty as to the nature of the quality of auditory hallucinations as they are experienced in psychosis (2a. Lack of Lived Experience). That the participants’ considerations may have been overly cautious due to a mixture of benevolent concern and a lack of informed knowledge must be acknowledged. An implicit assumption was present in the interviews that those with psychotic symptoms are inherently vulnerable. However, the literature on recovery would suggest that symptom reduction represents only a small portion of what being in recovery means for many with psychosis (Van Eck et al., 2018), with existential aspects such as agency and meaning often being far more salient for individuals (Lysaker et al., 2010; O’Keeffe et al., 2021; O'Keeffe et al., 2022). Given this, there is a clear potential for Patient-Public Involvement research (PPI) in the development of VR tools for psychosis research; the active collaboration of researchers and patients throughout the entire research process (Totzeck et al., 2024) and an importance to let those with psychosis speak on their own behalf.

These results additionally highlight the significance of the integrity of the research team (4c. The Responsibility of the Research Team). The acceptability of the VR-sAH was increased due to the researchers curating a safe and communicative environment in which to conduct the experiments. Although these results are broadly in line with past research highlighting the duty of care on the part of researchers (Madary & Metzinger, 2016; Rus-Calafell et al., 2018), it nonetheless solidifies the notion of ethical integrity being paramount to acceptability.

These results also point to subtle ways in which this VR tool may be improved. Although the Hearing Voices Simulation (HVS) was considered effective at creating the feeling of hearing voices due to stereo panning of the audio (2b Design Considerations), there is scope for using more culturally specific audio tracks in future research. The American voices of the HVS were considered by some to take them ‘out’ of the experience (2a. Lack of Lived Experience). Matching the voices to the nationality of the research base could increase validity. Furthermore, some participants were distracted by both the low visual quality of the VR tool and the crude fidelity of the avatars (2b Design Considerations), which is likely to diminish immersion (Christophers, Mulvaney & Rooney, 2024). Other published VR literature specifically notes that immersive VR enhances the feeling of presence needed for affective response (Diemer et al., 2015).

### Limitations

There are a number of methodological limitations. Firstly, for the psychophysiological results, there was no control measure used such as a VR condition without simulated hallucinations. Secondly, although the psychophysiology literature supports the use of ultra-short term recordings for HRV analysis (i.e., less than two minutes) and this research utilised recording durations that fell within the acceptable range, it should be noted that they are nonetheless considered proximal measures that attempt to estimate long term metrics (Shaffer, Meehan & Zerr, 2020). Thirdly, although saturation was reached during interviews, only a subset of the total participant sample conducted interviews. This implies that further investigation is needed as the limited sample restricts broad generalisability.

### Future directions: continued use in non-clinical populations

Our participants echoed the concerns of Madary & Metzinger (2016), who argue for care and due diligence to be taken on the part of the research team as to who experiences VR in psychological experiments. As such, these considerations infer that the development and application of VR-sAH should proceed slowly in relation to clinical populations. In the meantime, there is still much to be gained from recruiting non-clinical populations. Psychosis-like experiences exist on a spectrum across the entire population (Linscott & Van Os, 2013; Ward et al., 2020). Using non-clinical samples allows researchers to examine subclinical expressions of psychosis without the confounding influences of medication, hospitalisation, chronic illness, or diagnostic uncertainty (Mason, 2015). It also allows researchers to investigate immersion, affective salience, psychophysiological responding, and acceptability without exposing clinically vulnerable individuals to potentially distressing experimental paradigms prematurely. Furthermore, it offers practical advantages such as easier recruitment, greater sample diversity, and the ability to explore trait-level vulnerability that may precede or even predict clinical outcomes (e.g., Rauschenberg et al., 2021).

In this way, non-clinical VR research may contribute to the refinement of clinically relevant models of psychosis while simultaneously informing ethical safeguards required for future clinical implementation. Some cognitive models of psychosis (e.g., Freeman, 2016; Garety et al., 2001) predict that clinical and non-clinical groups with psychotic-like experiences will differ in how they respond to their anomalous experiences. However, the published data would indicate that the quality of auditory hallucinations does indeed change, with clinical and non-clinical groups being differentiated by increasingly hostile and malevolent voices (Larøi et al., 2012; Sorrell et al., 2010). It is also likely that affective states (or mood) play a large role in determining these differences (Scott et al., 2020). Thus, although certain aspects of models of psychosis may be tested using non-clinical populations, researchers should be aware that there may not be a 1:1 likeness in the quality of psychotic-like symptoms as they shift from non-clinical to the need for clinical care (as, for example, with the spectrum of paranoia described by Freeman, 2016). As such, the utility of using VR-sAH with non-clinical samples in tightly controlled experimental designs may add to the theoretical value in testing and building better models of the development of psychosis (Mason, 2015). However, as discussed, if non-clinical populations are to be used, careful screening is needed to minimise the risks of aggravating an existing psychological disorder or an undetected psychiatric vulnerability (Madary & Metzinger, 2016).

## Conclusion

These findings support the conclusion that the VR-sAH paradigm is both an acceptable and experientially valid research tool within a non-clinical population. More broadly, this study contributes to the development of ethically grounded reporting standards for immersive VR psychosis paradigms by demonstrating how acceptability may be systematically evaluated prior to implementation in clinical populations.

## Declaration of generative AI and AI-assisted technologies in the writing process

No AI was used in the process of this research.

## Ethics approval

This project received full ethical approval from University College Dublin’s Human Subjects Research Ethics panel (HS-24–90-OLeary-Gaynor).

## Consent to participate

Full informed consent to participate was acquired from all participants.

## Funding

This research did not receive any specific grant from funding agencies in the public, commercial, or not-for-profit sectors.

## CRediT authorship contribution statement

**Donagh Seaver O'Leary:** Conceptualization, Methodology, Validation, Formal analysis, Investigation, Writing – original draft, Writing – review & editing, Visualization, Project administration. **Pat Mulvaney:** Software, Visualization, Writing – review & editing. **Laura Moore:** Investigation. **Lera O’Connor:** Investigation. **Brendan Rooney:** Conceptualization, Methodology, Writing – review & editing. **Keith Gaynor:** Conceptualization, Methodology, Validation, Writing – review & editing, Supervision, Project administration.

## Declaration of competing interest

The authors have no competing interests to declare that are relevant to the content of this article.

## Appendix Supplementary materials

Image, application 1
