# Supplementary material for: Investigating the acceptability and validity of a novel VR paradigm that simulates auditory hallucinations
Source: Int J Clin Health Psychol. 2026 May 28;26(2):100694. doi: 10.1016/j.ijchp.2026.100694 (PMC13240750; doi:10.1016/j.ijchp.2026.100694)
Supplement: Supplementary file 1 [file mmc1.docx]

**SUPPLEMENTARY MATERIALS**

**A.**

**The Hearing Voices Simulation**

The hearing voices simulation, developed by Pat Deegan, is an audio track that simulates what it is like to hear audio-verbal hallucinations in psychosis. For more information and to hear a sample, see: <https://www.patdeegan.com/hearing-voices>.

**B.**

**Questionnaires**

Before the experimental trial, the CAPE-42 was administered to participants. The CAPE-42 (Stefanis  et al., 2002) is a widely administered self-report scale consisting of 42 items that assesses positive, negative, and depressive symptoms in relation to psychotic experiences in the general population. Meta-analytic studies indicate the CAPE-42 has high internal consistency and test-retest reliability (Cronbach's α > 0.80) across the three symptom dimensions (Mark & Toulopoulou, 2016). It has previously been used as a means to stratify non-clinical participants into high/low psychosis proneness (e.g., Counotte et al., 2017; Barbato et al., 2021). It uses a 4-point Likert scale (1 = Never, 2 = Sometimes, 3 = Often, 4 = Nearly always).

After the experimental trial, The Subjective Units of Distress Scale (SUD)(Wolpe, 1990) was administered. The SUD is a self-reported to assess a client's subjective level of emotional distress related to a specific memory, thought, or event. It allows for a quick, easy-to-understand, and non-intrusive method for assessing and monitoring distress. The visual SUD scale allows individuals to rate their distress on a horizontal scale from 0 to 100, with 0 representing complete calm or absence of distress and 100 representing the highest level of distress imaginable.

**C.**

**Experimental** **Procedure**

All experiments took place in the School of Psychology, University College Dublin between February-June 2025.

***ECG Set up***

Participants were first prepared for ECG recordings. Participants’ skin cleaned with an alcohol wipe. After ground strap was placed over their left wrist, each participant was then fitted with 4 electrodes (two on the front of each bicep, one positive and one negative). This electrode placement was chosen to mitigate ECG signal interference by the vibration of the EMDR pulsers. All physiological recordings were taken with participants seated in an upright posture (see Image 2).

Prior to the immersive VR experience, baseline ECG measures were taken for 2 minutes. As per recommendation by Quintana, Alvares & Heathers (2016), a ‘vanilla task’ was performed; a task that narrows attention but is valance-free and does not tax working memory. For the vanilla task during baseline recordings, participants watched a screen with a video of fish swimming around a fish bowl (commonly used in HRV analysis, e.g., Piferi et al., 2000). The video was sourced from open access internet repository ‘Internet Archive’ (Appendix Y). Participants sat 80cm from a 27” Full HD (1920x1080 pixels) monitor (ASUS, Taiwan).

**Image 2.**

*Photo of experimental set up for participants*

**
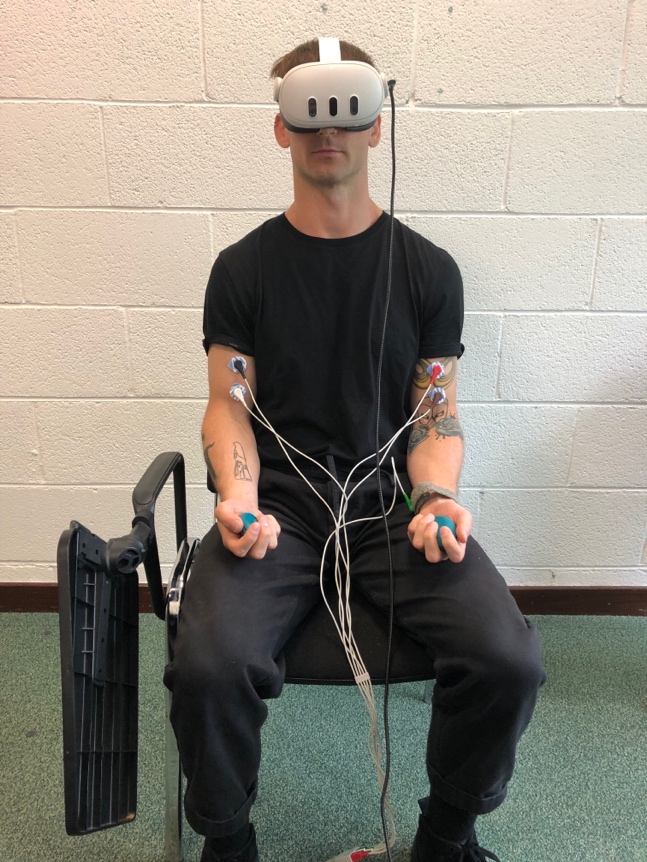
**

***VR***

Participants placed on the Meta Quest 3, adjusting as needed for comfort. Once they were ready, the immersive VR experience began.

***Post-VR***

Participants were helped with the removal of the VR headset and ECG electrodes. They then completed the SUD. Upon completion, all participants were issued with a full debrief, explaining the purpose of the research. Any questions that may have arisen were answered by the researcher.

**D.**

**Statistical Analysis and Data Preparation**

***Statistical Analysis***

All statistical analysis was undertaken using SPSS (IBM, version 29). Continuous outcome variables were checked for normal distribution using Shapiro-Wilks tests, the skewness and kurtosis of distribution, and by visually inspecting histograms and normal Q-Q- plots. Data were considered normal as assessed by Shapiro-Wilks, were less than ±1 kurtosis and skewness, and resembled reasonably normal distributions upon visual inspection of the histograms and normal Q-Q plots. Any non-normal data were log (ln) transformed. All log (ln) transformed data met assumptions of normality.

For all analyses, p-values <0.05 (two-sided) were considered statistically significant. Bonferroni corrections were employed in cases where multiple measurements were necessary. Due to the sample size, this study is only powered to detect medium to large effect sizes. Effect sizes were calculated using Quintana’s (2017) criteria, as HRV effect sizes appear to be different than standard Cohen’s *d*as used in other psychological research. To better reflect the observed effect size distribution, effect sizes of 0.25, 0.5, and 0.9 were used to be interpret small, medium, and large effects, respectively. Although Quintana (2017) does not specify HRV effect size norms in terms of partial eta squared, this research took into consideration the effect size distribution when interpreting magnitude using partial eta squared.

***Heart Rate Data Preparation***

Raw ECG signal files were analysed in Labchart (version 8 for Mac) using the ECG Analysis Module; an add-on that provides specialized tools for detecting and analysing PQRST components in ECG data. During recording, 2 channels of ECG data per participant were recorded. No data was lost during the experiments. For analysis, a single channel was chosen by inspecting the waveforms for the cleanest signal of the two.

The software algorithm automatically detected and categorised R peaks. All R peaks flagged by the classifier program were visually inspected by the researcher, and any beats incorrectly marked were manually adjusted. Labchart’s HRV Classifier plot was used to visually check for the presence of outliers. Artifacts - such as signal noise produced by movement during recording - were visually inspected at this time. Any missing beats were manually added. Where ambiguity occurred, R peaks were cross-referenced between the two channels. Where this was not possible, no R peak was marked. All ectopic beats were excluded from analysis.

Data were exported in a .txt file containing RR interval timings.

***Heart Rate Data Analysis***

The RR Interval .txt files were imported into Matlab (version R2024b, 2024, MathWorks Inc., Natick, Massachusetts) and HRV analysis was undertaken using Vollmer’s (2019) HRVTool, an open-source environment for HRV analysis in Matlab.

Because spectral HRV requires complete, uninterrupted RR data, any missing RR intervals were coded algorithmically in MatLab by computing the mean of the successive RR intervals. This was done for 32 of the total analysis files (representing 15.9% of the total 201 data files; 65 participants x 3 measures each). No file contained more than two successive missing RR intervals. No additional adjustments of beat locations was required. HRVTool automatically calculated HR and RMSSD scores for each section.
